# Further evidence of *Ceratobasidium* D.P. Rogers (Basidiomycota) serving as the ubiquitous fungal associate of *Platanthera leucophaea* (Orchidaceae) in the North American tallgrass prairie

**DOI:** 10.1186/s40529-020-00289-z

**Published:** 2020-04-15

**Authors:** Hana L. Thixton, Elizabeth J. Esselman, Laura L. Corey, Lawrence W. Zettler

**Affiliations:** 1 Department of Biology, Southern Illinois University Edwardsville, 1 Hairpin Dr., Edwardsville, IL 62025 USA; 2 Department of Biology, Illinois College, 1101 W College Ave., Jacksonville, IL 62650 USA; 3 Present Address: Department of Biology, West Virginia University, P.O Box 6057, Morgantown, WV 26506 USA

**Keywords:** Specificity, Conservation, Endophytes, Mycorrhizal fungi, *Tulasnella*

## Abstract

**Background:**

In the United States and Canada, ca. one-half of native orchid species are now threatened with extinction. A number of these species are restricted to tallgrass prairies of central North America, such as the Eastern Prairie Fringed Orchid, *Platanthera leucophaea* (Nutt.) Lindl.—a U.S. Federally threatened species.

**Results:**

We provide new records of fungi recovered from roots of *P. leucophaea* and five other orchid species inhabiting prairie sites in Illinois and neighboring states during a 10-year period (2008–2017). A total of 39 fungal endophytes were isolated from *Cypripedium candidum* (1), *Platanthera lacera* (1), *P. leucophaea* (32), *P. peramoena* (3), *Spiranthes lacera* (1), and *S. magnicamporum* (1), 31 (79%) of which were assignable to *Ceratobasidium* and the remainder to *Tulasnella*. These fungi were acquired from 16 different sites, 13 of which are new records including two new state records (Iowa, Wisconsin). Molecular analysis revealed that some *Ceratobasidium* strains were virtually identical despite being geographically isolated by > 300 km.

**Conclusions:**

This study, encompassing a decade of work, confirms that *Platanthera leucophaea* is a mycorrhizal specialist with heavy reliance on *Ceratobasidium* with the tallgrass prairie ecosystem of North America. Our isolation of *Ceratobasidium* from *P. leucophaea* spanning additional sites suggests that the association is widespread. Such information should provide conservationists and land managers with more confidence in developing protocols that facilitate the long-term conservation of this prairie orchid.

## Background

**Fig. 1:**
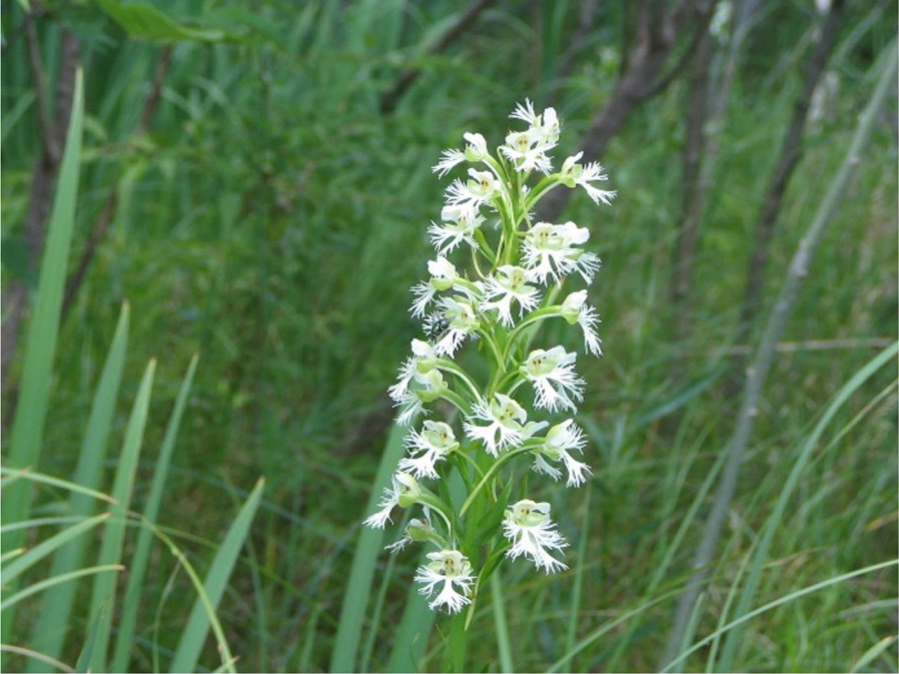
The U.S. Federally-listed (threatened) Eastern Prairie Fringed Orchid, *Platanthera leucophaea* (Nutt.) Lindl. (Photo courtesy of Dr. Timothy J. Bell)

In the United States and Canada, about one-half of native orchid species are now threatened with extinction (Krupnick et al. 2013). A number of these species are restricted to tallgrass prairies of central North America (Bowles 1983) including the Eastern Prairie Fringed Orchid, *Platanthera leucophaea* (Nutt.) Lindl. (Fig. 1). Millions of hectares of tallgrass prairies have been converted to agriculture resulting in isolated patches of prairie remnants prone to fire suppression, drainage and invasive Old World weeds (Ladd 1995). Today, only ca. 0.1% of original tallgrass prairie habitat remains (Samson and Knopf 1994; Leach and Givnish 1996), primarily in areas that were never tilled (e.g., cemeteries, railroad tracks), and occasionally in abandoned agricultural fields. These areas currently serve as ‘refugia’ for rare orchids and their associated biotic agents (e.g., pollinators, mycorrhizal fungi).

Studies researching fungi of orchids inhabiting tallgrass prairies date back to Curtis (1939) who described four ‘orchid species of *Rhizoctonia’* from roots of *Habenaria* (*Platanthera*) *leucophaea* in Wisconsin. More than 60 years later, other studies (e.g., Zettler et al. 2001) documented *Rhizoctonia*-like fungi from *P. leucophaea* in several Illinois prairie sites and one in Michigan, supporting Curtis’ early work. Most of the fungi recovered were identified as anamorphs in the genus *Ceratorhiza* using Moore’s (1987) classification system which has since been disbanded in favor of the teleomorphic equivalent, *Ceratobasidium* D.P. Rogers (1935). In addition to roots of mature plants, *Ceratobasidium* was also recovered with regularity from protocorms and seedlings of *P. leucophaea* (Zettler et al. 2001, 2005; Zettler and Piskin 2011), and several of these strains facilitated in vitro symbiotic seed germination. Thus, *P. leucophaea* appears to utilize *Ceratobasidium* fungi spanning the life of the orchid. *Ceratobasidium* has also been isolated with regularity from other prairie-inhabiting orchids such as *P. lacera* (Michx.) G. Don (L. Zettler, unpubl. data), *Spiranthes vernalis* Englemann and Gray (Morton 2015), and *P. leucophaea*’s ‘sister species’, *P. praeclara* Sheviak and Bowles (Sharma et al. 2003a, b).

Why *P. leucophaea* associates with *Ceratobasidium* is not known, but it is conceivable that the orchid targets this fungal genus to best meet its nutritional needs in the unique prairie ecosystem. Understanding the underlying mechanisms that favor the persistence of *Ceratobasidium* in North American prairies, at least in the few remnant fragments that remain, becomes crucial for orchid conservation in the region. In this paper, we provide new records of fungi recovered from *P. leucophaea* and five other orchid species inhabiting prairie sites in Illinois and neighboring states, expanding on a comprehensive study by Zettler and Piskin (2011). Fungi were provisionally identified to genus level using standard observational techniques (light microscopy, cultural characteristics). Molecular methods (ITS amplification and sequencing) were also used to confirm identification of select strains, and to detect genetic variation of *Ceratobasidium* strains within and between orchid populations.

## Methods

### Fungal collection, isolation and identification

During a 10 year period (2008–2017), living roots from 6 orchid species were collected from tallgrass prairie sites in four states (Illinois, Iowa, Michigan, Wisconsin) during the growing season (June–August). These species consisted of *Cypripedium candidum* Mühl. ex. Willd., *Platanthera lacera* (Mich.) G. Don, *P. leucophaea* (Nutt.) Lindl., *P. paramoena* (Gray) Gray, *Spiranthes magnicamporum* Sheviak, and *S. vernalis* Emgl. and Gray. Lateral roots were detached from donor plants, placed in plastic Ziploc^®^ bags (Dow Chemical Co.) along with soil, and transported to the laboratory within 24 h. Fungi were isolated from roots using the procedures outlined by Zettler and Corey (2018). This process consisted of surface-sterilizing the roots using a sterile solution of 5 ml Clorox^®^ bleach (5.25% NaOCl, Clorox Co., Oakland, CA), 5 ml absolute ethanol, and 90 ml sterile DI water. Roots were then rinsed twice with sterile DI water, segmented into 1 mm pieces and placed into separate Petri plates. The segments were macerated thoroughly in a drop of DI water to tease out pelotons from within the root cortex. Molten Fungal Isolation Medium (FIM; Clements et al. 1986) was poured into plates and gently swirled to space the pelotons apart prior to the agar’s solidification. Plates were incubated for 24–48 h at ambient temperature until visible signs of hyphae could be seen growing into the agar. Assisted by a dissection microscope, hyphal tips were removed from fungal colonies using a sterile scalpel and placed onto the surface of Potato Dextrose Agar (PDA, Difco™, Becton–Dickinson and Co., Sparks, MD). After 1–2 weeks of incubation at ambient temperature, fungal colonies were inspected visually and microscopically. Those that matched published descriptions of fungi in the *Rhizoctonia* complex (Currah et al. 1997; Zettler and Corey 2018) were retained for further study. Several of these isolates were selected for further identification using molecular (PCR) methods.

### Molecular identification

Molecular identification followed the procedures outlined by Zettler et al. (2013) and Yokoya et al. (2015) involving ribosomal DNA internal transcribed spacer (ITS) amplification and Sanger sequencing. A 1 cm^3^ of fungal inoculum from each culture was added to a flask containing potato dextrose broth (PDB; Sigma Aldrich, St. Louis, MO) and the flask was gently swirled on a shaker for ca. 3 weeks (21–30 days) at ambient temperature until globular fungal colonies measuring ca. > 5 cm diam. were visible in the broth. The Extract-N-Amp™ Plant PCR Kit (Sigma Aldrich) and protocol were used and followed to extract and amplify the fungal DNA from the fungal colonies. DNA primers for amplification and PCR conditions were chosen carefully from White et al. (1990) and Taylor and McCormick (2008) based on what was concluded to work best for the two primary genera of *Rhizoctonia*-like fungi (*Ceratobasidium*, *Tulasnella*). DNA samples that were not used immediately were stored at − 20 °C; however, after multiple amplification attempts, it became clear that the stored DNA samples had decreased in quality and would no longer support PCR amplifications. DNA quality was checked for each sample (gel electrophoresis on a 1.5% agarose gel at 150–175 milliamps for 15–20 min) and simultaneously quantified using the Nanodrop^®^ (Thermo Scientific) spectrophotometer. Gels stained with ethidium bromide were photographed with a BIO-RAD ChemiDoc™ MP Imaging System. After extracting DNA from the fungal samples, amplification reaction of 20 µl was conducted using primers ITS 1 and 4 or CeTh1 and CeTh4 (White et al. 1990; Taylor and McCormick 2008) selected based on the findings of White et al. (1990) and purchased from Integrated DNA Technologies (Skokie, IL USA). PCR was using JumpStart Taq Ready Mix from Sigma (St. Louis, MO) and final primer concentrations of 0.4 pmol/µl. PCR was carried out in a LabNet™ thermocycler with the following conditions: 94° for 5 min, then 35 cycles of 94° for 30 s, annealing temperature 48° (ITS1 and 4) or 57° (CeTh1 and CeTh4) for 30 s, then 72° for 30 s, followed by a final extension of 72° for 10 min. After the PCR amplification, the products were visualized through gel electrophoresis as described for the fungal genomic DNA. Successfully amplified DNA was cleaned, and the PCR products were sent to the Core DNA Sequencing Facility at the University of Illinois, Urbana-Champaign. Sample preparation for submission followed their guidelines available at https://unicorn.biotech.illinois.edu/-. An NCBI (National Center for Biotechnology Information) BLAST search was performed on GenBank to identify possible matches to the fungal sequences. Consensus sequences were determined from reactions using the forward and reverse primers and were aligned with their top matches from the BLAST (blastn) search (Altschul et al. 1997; Taylor and McCormick 2008).

## Results

**Fig. 2:**
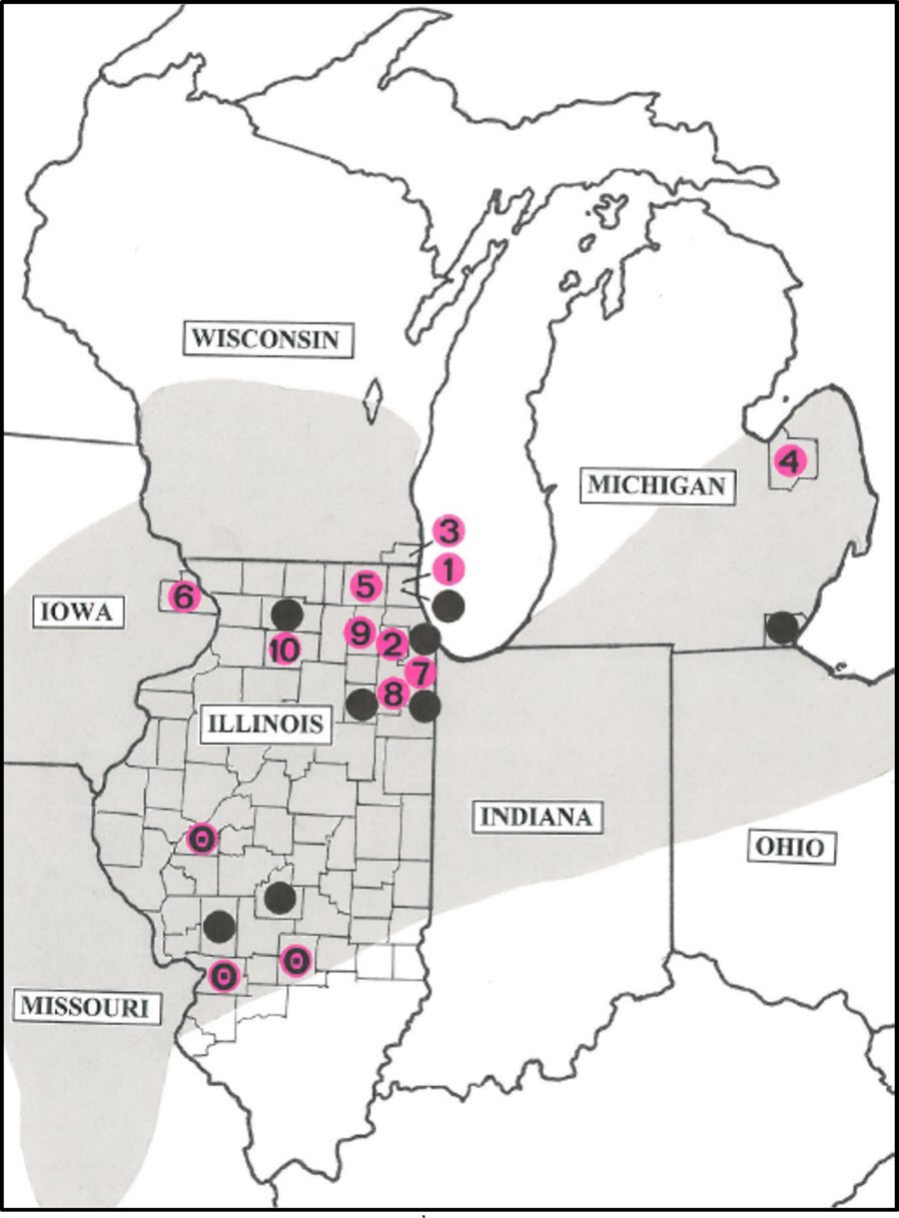
Locations in the Midwestern United States where *Ceratobasidium* fungi were isolated from roots and/or protocorms of *Platanthera leucophaea* as of 2017. The shaded areas reflect the orchid’s historic distribution (Bowles 1983). Solid dots indicate locations where *Ceratobasidium* (=*Ceratorhiza*) was recovered from *P. leucophaea* prior to the present study. The two solid dots from lower region of Illinois (Christian and Macoupin Co.) were locations where protocorms of *P. leucophaea* were acquired from seed packets (see Zettler and Piskin 2011). Pink dots with numbers represent sites where *Ceratobasidium* was acquired from *P. leucophaea* in the present study: 1 = Lake Co., IL; 2 = Dupage Co., IL; 3 = Kenosha Co., WI; 4 = Tuscola Co., MI; 5 = McHenry Co., IL; 6 = Jackson Co., IA; 7 = Cook Co., IL; 8 = Will Co., IL; 9 = Kane Co., IL; 10 = Lee Co., IL. Pink dots lacking a number reflect tallgrass prairie sites where *Ceratobasidium* was isolated from orchids other than *P. leucophaea* (i.e., *Platanthera lacera*, *P*. *peramoena*, *Spiranthes magnicamporum*, *S. vernalis*)

During the 10-year period (2008–2017), a total of 39 new records of fungal endophytes were recovered from six native orchid species from tallgrass prairies in the United States in Illinois, Iowa, Michigan, and Wisconsin, mostly from roots of *Platanthera leucophaea* in Illinois where the species is most abundant (Table 1, Fig. 2). Of these 39 isolates, 31 (79%) were assignable to the genus *Ceratobasidium*, and the remainder to *Tulasnella*. These fungi were acquired from 16 different sites, 13 of which are new records including two new state records—Iowa (Baldwin Marsh, Jackson Co.) and Wisconsin (Chiwaukee, Kenosha Co.). Of the few (eight) *Tulasnella* endophytes recovered, six were from *P. leucophaea* in Bystricky Prairie (McHenry Co., IL) and five of these originated from different juvenile plants spanning 3 years (2012–2014). The other two *Tulasnella* strains were documented from Sundrop Prairie (Cook Co., IL) and Horn’s Prairie (Fayette Co., IL), from roots of mature *P. leucophaea* and *P. peramoena*, respectively (Table 1). Three of the six orchids studied were *Platanthera* species (*P. lacera, P. leucophaea*, *P. peramoena*) and the other three included *Cypripedium candidum* and two species of *Spiranthes* (*S. magnicamporum*, *S. vernalis*). To our knowledge, this is the first published report documenting fungal endophytes from *P. peramoena* in North America.

**Table 1:** A summary of the orchid endophytes recovered from orchids inhabiting tallgrass prairies during the past 10 years (2008–2017)

Year	State	County	Population	Orchid	Growth/Plant ID#	Fungus	ID#	Acc. #
2008	IL	Cass	Rexroat Prairie	Sp mag	M	C	IC 350	–
2011	IL	Lake	Wrigley-Abbott	P leu	M (#5588)	C	IC 356	–
2011	IL	Lake	Wrigley-Abbott	P leu	M (#5509)	C	IC 357	–
2011	IL	Lake	Wrigley-Abbott	P leu	M (#5568)	C	IC 360 UAMH 11548	–
2011	IL	Lake	Wrigley-Abbott	P leu	M (#5569)	C	IC 361	–
2011	IL	Dupage	Swift Prairie	P leu	M (#5291)	C	IC 358/359 UAMH 11546	–
2011	WI	Kenosha	Chiwaukee	P leu	M	C	IC 362	–
2011	WI	Kenosha	Chiwaukee	P leu	M	C	IC 363 UAMH 11573	–
2011	IL	Lake	Lyon’s Woods	P leu	M (#11087)	C	IC 365	JS546242
2011	IL	Lake	Lyon’s Woods	P leu	M (#11126)	C	IC 366/367 UAMH 11576	–
2011	IL	Cook	Sundrop Prairie	P leu	M	T	IC 368/369	–
2011	MI	Tuscola	Unionville FWS	P leu	M	C	IC 370 UAMH 11579	DQ068771
2012	IL	McHenry	Bystricky	P leu	M (#7840)	T	IC 372	–
2012	IL	McHenry	Bystricky	P leu	M (#7804)	C	IC 373	–
2013	IL	McHenry	Bystricky	P leu	J (#8304)	T	IC 386	–
2013	IL	McHenry	Bystricky	P leu	J (#7890)	C	IC 387	–
2013	IL	McHenry	Bystricky	P leu	J (#7621)	C	IC 388	–
2014	IL	Fayette	Horn’s Prairie	P lacera	M	C	IC 413/414	–
2014	IL	McHenry	Bystricky	P leu	J (#7841)	T	IC 417	JQ247553
2014	IL	McHenry	Bystricky	P leu	J (#7627)	T	IC 418	–
2015	IL	McHenry	Bystricky	Cyp can	M	T	IC 419	–
2015	IL	Madison	SIUE-Poag Rd	Sp vern	M	C	IC 423 SIUe 9-B	–
2015	IL	Fayette	Horn’s Prairie	P peram	M (#1)	C	IC 424 SIUe L2CA	MG662854
2015	IL	Fayette	Horn’s Prairie	P peram	M (#1)	T	IC 425 SIUe L2AE	AY373273
2015	IL	Fayette	Horn’s Prairie	P peram	M	C	IC 426 SIUe L1D-0	JX546237
2016	IA	Jackson	Baldwin Marsh	P leu	M (#158)	C	SIUe HT100, (101,103,104)	JF912404
2016	IA	Jackson	Baldwin Marsh	P leu	M (#158)	C	SIUe HT105, M114	MG662854
2016	IA	Jackson	Baldwin Marsh	P leu	M (#158)	C	SIUe HT116	DQ068771
2016	IL	Cook	Helm Road	P leu	M (#5172)	C	SIUe HT102	AF504008
2017	IL	Will	Grant Creek	P leu	J (#3)	C	SIUe HT157	MG663053
2017	IL	Will	Grant Creek	P leu	J (#2)	C	IC 430 SIUe HT148	–
2017	IL	Kane	Lone Grove	P leu	J (#2)	C	SIUe HT122	MG662974
2017	IL	Lee	Nachusa	P leu	J (#3)	C	SIUe HT135	AF504008
2017	IL	Cook	Helmroad	P leu	J (#1)	T	SIUe HT170	JQ247568
2017	IL	Cook	Helmroad	P leu	J (#3)	C	SIUe HT169/198	AF504008
2017	IL	Cook	Helmroad	P leu	J (#3)	C	SIUe HT196	MG663023

Fungi denoted by C = *Ceratobasidium*, T = *Tulasnella*

ITS amplification and Sanger sequencing confirmed that all strains collected in 2016 and 2017 were assignable to *Ceratobasidium* (Table 1). Molecular analysis also revealed that genetically different strains of *Ceratobasidium* were detected in one specific individual orchid, evidenced by the *P. leucophaea* sample collected in 2016 from the site in Iowa (Baldwin Marsh) that yielded three different strains from a single mature plant (#158; Table 1). This observation was also noted for a juvenile *P. leucophaea* specimen collected in 2017 in Illinois (Helm Road) that harbored two different *Ceratobasidium* strains (Table 1). When different strains were genetically analyzed and compared via UPGMA phenogram, virtually identical strains of the same fungus were prevalent in geographically distant regions. For example, *Ceratobasidium* IC 370 from Michigan (Unionville, Tuscola Co.) displayed nearly identical matches to a strain collected from *P. praeclara* in NCBI’s GenBank repository (Accession number MG662854.1l Benson et al. 2017; Table 1). Moreover, *Ceratobasidium* IC 365 from a *P. leucophaea* specimen in Lake Co., Illinois (Lyons Woods) was virtually identical to a strain (IC 426) recovered from *P. peramoena* in Fayette Co. (Horn’s Prairie) > 300 km to the south (Table 1).

## Discussion

Compared to other North American orchid species, a modest amount of research has been published with respect to *Platanthera leucophaea* including its habitat, pollinators, seed germination requirements, and in vitro propagation (e.g., Bowles 1983; Bowles et al. 2002, 2005; Pollack 2009; Stoutamire 1996) in addition to its mycorrhizal associates (e.g., Zettler et al. 2001, 2005). Prior to this study, Zettler and Piskin (2011) documented 75 orchid endophytes in protocorms, seedlings, and roots from mature *P. leucophaea* collected in Illinois and Michigan, 66 of which (88%) were identified as *Ceratorhiza* (=*Ceratobasidium*), and the remainder were *Epulorhiza* (=*Tulasnella*). This study confirmed the presence and persistence of *Ceratobasidium* in three of the Illinois prairies they reported: Wrigley/Abbott (Lake Co.), Nachusa (Ogle Co.) and Grant Creek (Will Co.). Collectively, 97 of 114 (85%) of the fungi isolated during a 20-year period by both studies were identified as *Ceratobasidium*, reaffirming that this genus is the dominant endophyte of *P. leucophaea* in the eastern region of the tallgrass prairie expanse (Fig. 2). Whether or not other co-habiting orchid species utilize *Ceratobasidium* to a large extent remains to be determined, but the ratio of *Ceratobasidium* to *Tulasnella* isolated from all orchid species in the present study was roughly 5 to 1 in favor of the former. In prairies west of the Mississippi River, *Ceratobasidium* has also been isolated with regularity from roots of the Western Prairie Fringed Orchid, *Platanthera praeclara* Sheviak and Bowles—the ‘sister species’ of *P. leucophaea* (Sharma et al. 2003a, b). Interestingly, Morton (2015) determined that a *Ceratobasidium* endophyte isolated from *Spiranthes vernalis* in Madison Co., Illinois had a 99% identity match to one obtained from *P. praeclara* in Clay Co., Minnesota—a distance separated by > 1000 km. Davis et al. (2015) reported that the mycorrhizal fungi utilized by an Australian orchid (*Pheladenia deformis*) belonged to a single OTU of *Sebacina* (with one exception) that was present on both sides of the continent (> 2000 km apart). These studies raise the prospect that *P. leucophaea* and perhaps other orchids of the tallgrass prairie may be mycorrhizal ‘specialists’ (Swarts and Dixon 2009) that are intimately tied to a narrow group of *Ceratobasidium* fungi to meet their mycotrophic needs. Indeed, Curtis (1939) predicted that an orchid species restricted to a specific habitat would likely harbor fewer types of fungal associates. The recovery of *Ceratobasidium* from early growth stages as well as mature plants of *P. leucophaea* (Zettler et al. 2005; Zettler and Piskin 2011) lends support for this fungal genus serving as the primary mycorrhizal associate (resident) from germination to anthesis. This hypothesis is further strengthened by Thixton (2017) who germinated seeds of *P. leucophaea* inoculated with three of the *Ceratobasidium* strains reported herein (IC 370, SIUe HT101-103). New studies are being planned that will test the other 26 fungal isolates (Table 1) for their ability to germinate *P. leucophaea* seeds in vitro. Plans are also underway to isolate and safeguard mycorrhizal fungi from additional sites throughout the region (e.g., central Wisconsin) and compare these fungi using molecular techniques. Of particular interest would be to isolate and compare fungi from nutrient-rich Wisconsinan-aged soils to more acidic nutrient poor soils of the Illinoian-aged drift outlined in Bowles et al. (2005).

Another area of interest would be to study the underlying mechanism(s) behind this close association linked to soil. Tallgrass prairies in the Midwest differ from other orchid habitats in two major respects—they are often regularly burned, and many are surrounded by agricultural land subject to chemical application (e.g., fertilizers). Both practices could conceivably alter soil nutrient levels in ways that affect a range of subterranean lifeforms (e.g., orchid protocorms, mycorrhizal fungi). Leff et al. (2015), in fact, noted that elevated N and P input led to a shift in microbial communities in grasslands, including mycorrhizal fungi. Unlike most other plants, orchids generally prefer organic forms of N to inorganic forms (Rasmussen 1995), and inorganic N may actually inhibit growth or lead to toxicity (Crawford 1995; Dechorgnat et al. 2011). Additionally, Figura et al. (2019) determined that orchids inhabiting oligotrophic habitats where highly sensitive to nitrate levels compared to orchids inhabiting eutrophic habitats which were largely insensitive. They also linked nitrate levels to orchid distribution because nitrate had a direct role on inhibiting seed germination. Less is known about the role of nutrient sources (mineral or organic) on orchid mycorrhizal fungi, and such information might help resolve whether *Ceratobasidium* is more prevalent in *P. leucophaea* roots simply because this fungus is more abundant in soil (M. Bowles, pers. com.). Hadley and Ong (1978) reported that isolates of *Ceratobasidium cornigerum* grew well on nitrate and other nitrogen sources and were more efficient than *Tulasnella calospora* in their nitrogen ‘economy’. More recently, Nurfadilah et al. (2013), explored the impact of potential carbon (C), nitrogen (N) and phosphorous (P) sources on the growth of *Ceratobasidium*, *Sebacina*, and *Tulasnella*—the three primary genera of mycorrhizal fungi in photosynthetic orchids—and demonstrated in vitro that all three fungal genera exploited a ‘wide and variable menu’ of C, N and P sources. They also determined that these fungi utilized ammonium as the source of inorganic N.

Given that *P. leucophaea* was historically more widespread prior to the conversion of tallgrass prairies to agricultural land, the role of frequent burning might be of particular interest with respect to its impact on the distribution and persistence of mycorrhizal fungi. Ramsay et al. (1986), determined that the abundance and composition of orchid mycorrhizal fungi is influenced by fire in SW Australia, and Bell et al. (2016), concluded that both fecundity and survival of *P. leucophaea* was higher in habitats that were burned. We urge future researchers to also consider examining the role of frequent burning on the prevalence and distribution of orchid mycorrhizal fungi in the tallgrass prairie. Analytically, this might be accomplished by testing various soil types for the prevalence of *Ceratobasidium*, for example. Experimentally, field tests could be carried out that compare growth and survival of transplanted *P. leucophaea* seedlings in burned versus unburned soils, and by in vitro studies that inoculate test soils with fungi and seeds.

## Conclusions

This study, encompassing a decade of work, confirms that *Ceratobasidium* serves as the primary mycorrhizal associate of *Platanthera leucophaea*. Such information serves as a useful baseline for long-term efforts aimed at the orchid’s survival in this ‘age of extinction’. According to Swarts and Dixon (2009), the ability to conserve terrestrial orchids this century will depend on three actions: (1) management of natural reserves, taking into account the specific needs of the orchids (e.g., mycorrhizal fungi, pollinators), (2) establishing seed and fungal storage ‘banks’, and (3) developing techniques for orchid restoration. The success of all three actions hinges, at least in part, on knowing more about the fungal associates within the root systems of the orchid and in the landscape. Our isolation of *Ceratobasidium* from *P. leucophaea* spanning additional sites suggests that the association is widespread. Such information should provide conservationists and land managers with more confidence in developing protocols that align with the guidelines proposed by Swarts and Dixon (2009).

## Data Availability

The authors confirm that local and national guidelines and legislation are followed by acquiring appropriate permissions and licenses for the study.
